# Antimicrobial, Antiradical Activity, and X-Ray Fluorescence Spectroscopy Analysis of *Aloe otallensis* Plant Used in Traditional Medicine in Southern Ethiopia

**DOI:** 10.1155/2024/1981990

**Published:** 2024-08-12

**Authors:** Yonas Syraji, Mulugeta Kebebew, Yohannis Techane, Dawit Albene

**Affiliations:** Department of Biology College of Natural Sciences Arba Minch University, Arba Minch, Ethiopia

## Abstract

Medicinal plants have a long history of treating diseases in animals and humans in Ethiopia. Nevertheless, not enough research has been done on the antibacterial properties and possible bioactive components of the majority of medicinal plants. Therefore, this study was concerned with the evaluation of the percentage yield, phytochemical, antimicrobial, antifungal, MIC, antiradical activities, phenolic content, and X-ray fluorescence spectroscopy (XRF) analysis of *A. otallensis* plant extracts. The mean values of antimicrobial, antifungal, MIC, antiradical, phenolic content, and XRF analysis were reported as mean ± standard deviation. The solvent methanol showed a higher degree of yield in leaf and root extract which was 8.45 (22.27%) and 3.12 g (15.58%), respectively, while distilled water extract of leaf and root showed less degree of yield which was 0.22 g (1.10%) and 0.42 g (2.1%), respectively. Qualitative phytochemical analyses of the plant parts have revealed the presence of various components of metabolites like alkaloids, flavonoids, phenol, saponins, tannins, steroids, steroids, terpenoids, triterpenoids, glycosides, anthraquinones, diterpenes, phytosterols, and phlobatannals. *A. otallensis* gel extracts had shown significant antibacterial and antifungal activity against the test bacterial and fungus, respectively. Moreover, the methanolic gel extracts of *A. otallensis* demonstrated notable antiradical activity than the leaf and the root. The highest value of phenolic content was obtained in *A. otallensis;* gel, leaf, and root extract which was 61.9 ± 0.5 mg/g, 53.6 ± 0.3 mg/g, and 51.6 ± 0.6 mg/g, respectively. In this study, twelve elements in the plant parts of *A. otallensis* were determined using XRF spectroscopy. Overall, this research contributes to the understanding of the pharmacological potential of *A. otallensis* and highlights the importance of further research into its medicinal properties. The results provide valuable insights into the use of medicinal plants to treat diseases and support the development of natural therapeutics.

## 1. Background

Ethnotherapeutic practice of plant species are believed to be one of the potential bases for the development of safe and effective treatments [1]. Ethiopia has a long history of a traditional health care system, but studies on traditional medicinal plants have been limited in comparison to the country's multiethnic, cultural, and flora diversity [2]. Moreover, the use of medicinal plants to treat infections is an old practice in large parts of Ethiopia to solve health problems for livestock and humans [3–5]. Yet, most of these experiences are not documented and supported by scientific experiments. Besides, antimicrobial resistance is spreading at an alarming rate, making treating bacterial infections in animals and humans more difficult. Even modern medicines have failed to treat those resistant bacteria since they are developing resistant behavior [6]. Though just 15% of the world's higher plants have been thoroughly studied for bioactivity, it makes sense to look for alternate approaches to treating infectious diseases. Additionally, plants have the potential to yield innovative antibiotics and medications [7].

The genus Aloe had recorded history of mentioned and given high rank as multipurpose herbal plant. It was renowned for its medicinal and cosmetic properties that have been exploited over millennia in Ethiopia [8, 9]. Recent ethnobotanical studies have been reported the use of various Aloe species as traditional medicines in Ethiopian communities to treat various ailments like malaria, stomachache, diarrhea, gonorrhea, impotency in men, anthrax, internal parasite, diabetes, liver disease, wounds healing, and skin infection [9, 10].

*A. otallensis* has also been used as a folk remedy to treat various ailments such as malaria, leishmania, worms and intestinal parasite, snake bite, and wound healing. It has shown antioxidant, antimalarial, and antileishmania properties [11, 12]. This is due to the presence of diverse and unique phytochemical compounds like anthrones, chromones, pyrones, coumarins, alkaloids, glycoproteins, naphthalenes, anthraquinones, flavonoids, and phenols [13, 14].

Despite the abovementioned medicinal uses of *A. otallensis,* only the antimalarial and antileishmania activity of the plant has been investigated with results showing genuine antimalarial and antileishmania activity [11, 12]. Many in-vitro investigations have been conducted, and the results have demonstrated the antibacterial properties of herbal remedies that have been traditionally used in different parts of Ethiopia. Nonetheless, the antibacterial qualities of some Ethiopian medicinal herbs are still awaiting scientific confirmation. Yet, no study has been carried out to investigate the antimicrobial activity, antiradical activities, and XRF analysis of *A. otallensis* plant extracts. In this study, we aim to comprehensively evaluate the antibacterial, antifungal, and antiradical activities of *A. otallensis* plant extracts using in-vitro assays. Additionally, we will conduct XRF analysis to characterize the elemental composition of the extracts. By elucidating the pharmacological properties and chemical composition of *A. otallensis* extracts, this research seeks to provide valuable insights into their therapeutic potential and contribute to the development of novel healthcare interventions.

## 2. Materials and Methods

### 2.1. Collection and Identification of the Plant Material

Fresh whole parts of *A. otallensis* (i.e., leaf, gel, and root) used for this experiment were collected following standard botanical procedures in February 2023. Plant materials and *A. otallensis* plant parts were collected from Yetnebershi which is found in Arba Minch, Ethiopia. Arba Minch is located at 6° 2″ *N* latitude and 37° 33″ E longitude, far about 500 km from Addis Ababa and at an elevation of 1285 m. Current estimated number of the zone total population (permanent residents) is 95373 people, including children under the age of 6–9478 people, teenagers (school children) aged 7 to 17 years–11314 people, young people from 18 to 29 years old–11385 people, adults aged 30 to 60 years–41070 people, elderly people over 60 years old–20791 people, and the centenarians Arba Minch over 80 years old–1335 people. Its total area has been estimated 10,000 km^2^ lying within an elevation of 710 to 4200 m above sea level. The identification of the plant sample was authenticated at Arba Minch University, College of Computational and Natural Sciences, Department of Biology.

### 2.2. Preparation of Plant for Extraction

The fresh leaves of *A. otallensis* were well washed with tap water, dried at 20–25°C, and grinded, respectively. The fresh roots of *A. otallensis* were also washed with tap water, dried at 20–25°C, and grinded, respectively. The fresh leaf of *A. otallensis* was cleaned with tap water, and then gel was extracted from the inner leaf pulp and homogenized aseptically using a sterile meal grinder [IKA.A11 BASIC, D-79219 Staufen]. The homogenized gels were squeezed using sterile cheesecloth. The filtrate was kept in 15 ml of test tubes and was stored at 20°C until use. Following that, 300 mg/ml of stock solution was prepared with 10% and 10 ml of DMSO solution which was considered as 100% in concentration.

### 2.3. Filtration, Evaporation, and Yield of Extracts

The extracts were filtered using Whatman No. 1 filter paper, the filtered extracts were concentrated by using a rotary evaporator, and the residual extracts were dried. The percentage yield was obtained using dry weight, from equation (1). The extracts were kept and stored in the refrigerator at 5°C until use.

(1) $$\begin{array}{c} \%\text{ Yield of extract }\left(\frac{g}{100}g\right)=\frac{\left(W1\times 100\right)}{W2}, \end{array}$$

where *W*1 is the weight of the extract residue after solvent removal and *W*2 is the weight of dried plant powder.

### 2.4. Preliminary Qualitative Phytochemical Screening of Plant Specimen

In different conical flasks, 5 g of powdered leaf and root sample was soaked for 72 hr with 50 ml of methanol, n-hexane, petroleum ether, and distilled water. A phytochemical screening method was used to identify and evaluate the presence of secondary metabolites, including alkaloids, flavonoids, phenol, saponins, tannins, steroids, terpenoids, triterpenoids, glycosides, anthraquinones, diterpenes, phytosterols, and phlobannals. The extracts were filtered. For the crude extracts of methanol, n-hexane, petroleum ether, and distilled water, a phytochemical screening was done. Standard protocols were followed in the screening process to look for secondary metabolites [15, 16].

#### 2.4.1. Test for Alkaloids

2 ml of aqueous extracts in the test tube was mixed with 2 drops of 1.5% HCl and filtered. 2 ml filtrate of plant drug extract and 2 ml of Wagner's reagent were mixed. Formation of reddish brown precipitate indicated the presence of alkaloids [17].

#### 2.4.2. Test for Flavonoids

1 ml of 10% lead acetate was added to 1 ml of the extract contained in a test tube. The formation of yellow precipitate was considered positive for flavonoids [18].

#### 2.4.3. Test of Phenols

1 ml of the aqueous extract and 3-4 drops of 5% FeCl_3_ (w/v) were added. Formation of the bluish black color indicates the presence of phenol.

#### 2.4.4. Test of Saponins

3 ml of the extract in a test tube was mixed with 5 ml of distilled water and shaken vigorously for 2 minutes. The formation of stable form or froths established the presence of saponins [19].

#### 2.4.5. Test for Tannins

2 ml of extract was taken in a test tube, and 3-4 drops of 5% ferric chloride solution were added, the formation of dark blue, which indicated the presence of tannins [16].

#### 2.4.6. Test for Steroids

A mixture of 1 ml of methanolic extract, 1 ml of chloroform, 2-3 ml of acetic anhydride, and 1-2 drops of concentrated H_2_SO_4_ was added. When 3 drops of concentrated H_2_SO_4_ were added to 3 ml of the extract, red coloration was observed, and this indicates the presence of steroids [19].

#### 2.4.7. Test for Terpenoids

A test for terpenoids was conducted by mixing 3 ml of extract with 2 ml of chloroform in a test tube. Thereafter, 2 ml of concentrated H_2_SO_4_ was added gently to form a ring layer which interfaced with a reddish brown color, thus indicating the presence of terpenoids [19].

#### 2.4.8. Test for Triterpenoids

Qualitative investigation of triterpenoids was followed according to the Libermann Buchard test. That is 1 ml of the crude extract was treated with 2 ml of acetic anhydride and boiled and cooled, and 2 ml concentrated H_2_SO_4_ was added from the side of the test tube. Formation of red colour solution indicated the presence of triterpenoids.

#### 2.4.9. Test for Glycosides

1 ml glacial acetic acid was added and left to cool down. After cooling, two drops of FeCl_3_ were added and 2 ml of concentrated H_2_SO_4_ along the walls of the test tube was dispensed carefully. Development of the reddish brown-colored ring at the intersection of two layers indicated the presence of glycosides [18].

#### 2.4.10. Test for Anthraquinones

Borntrager's test: 3 ml of aqueous extract was shaken with 3 ml of benzene; filter and 5 ml of 10% ammonia solution was added to the filtrate. The mixture was shacked, and the presence of a pink, red, or violet color in the ammonical (lower) phase indicates the presence of free anthraquinones [19].

#### 2.4.11. Test for Diterpenes

2 ml of extracts was dissolved in 2 ml of distilled water and treated with 3-4 drops of copper acetate solution. Formation of emerald green color indicates the presence of diterpenes.

#### 2.4.12. Phytosterols

2 ml of extracts were dissolved in 3 ml of the chloroform filter and treated with 3-4 drops of H_2_SO_4_ solution. Formation of yellow indicates the presence of phytosterols.

#### 2.4.13. Phlobatannins

1 ml of the extract was dissolved in 1 ml of HCl solution boiled. Formation of the red precipitate indicates the presence of phlobatannins.

### 2.5. Evaluation of Antibacterial Activities

#### 2.5.1. Tested Microorganisms

A total of two bacterial and one fungus were used in this experiment for the zone of inhibition and minimum inhibitory concentration (MIC) assays. The bacteria were obtained from the Ethiopian Public Health Institute (EPHI), Addis Abeba, Ethiopia. The bacterial strains under this study include *Escherichia coli* ATCC25922 and *Staphylococcus aureus* ATCC25923. The fungus used in this study was dandruff. Bacterial isolates were maintained at 2 and 8°C on nutrient broth, while the fungal isolates were maintained at 4°C on Sabouraud dextrose agar (SDA) media.

#### 2.5.2. Inoculum Preparation and Preparation of Test Solutions

The tested microorganisms were separately cultured on sterilized Muller–Hinton agar (MHA) at 37°C for 24 hr by using the streak plate method. Then, well-isolated overnight cultured colonies of the same morphological type were selected from the cultured media. Each colony was touched with a flamed wire loop, and the growth was transferred into a sterilized test tube containing 5 ml sterile normal saline solution. The test tubes that contain the bacterial suspension were vortexed to be mixed well uniformly. Then, the bacterial suspension was adjusted with 0.5 McFarland turbidity standards. The adjustment and comparison of turbidity of inoculum tubes were performed by using spectrophotometer reading against 0.5 McFarland turbidity.

#### 2.5.3. Assay of Antibacterial Activity by Agar Well Diffusion Method

The agar well diffusion method expresses the results as the width of the inhibition zone produced by the plant extract [20]. The plant extracts were tested against bacterial strains. For the agar well diffusion method, 5 mm size well was prepared in the cultural strain swabbed plates with the help of a well cutter. Then, 5 *µ*l of plant extracts were added to the well by using a micropipette. 5% of dimethyl sulfoxide (DMSO) was carefully added to the well as a control. Then, plates were incubated at 37°C for 24 hr. After the incubation period, the zone of inhibition was measured. A well was prepared in the plates with the help of a cork-borer (5 mm) [21].

### 2.6. Determination of Minimum Inhibitory Concentration (MIC) of Plant Extract by Agar Dilution Method

The MIC of all crude extracts was evaluated against *S. aureus* and *E. coli*. A 5% DMSO was used to dilute crude plant extracts. Then, after serial dilution, the crude extract of 2 ml was mixed with molten MHA 18 ml and poured into sterilized Petri dishes. The plate was inoculated with the standardized (0.5 McFarland standard) bacterial inoculum and incubated at 37°C for 24 hr. The result of bacterial inhibition was judged by comparison with growth in positive and negative controls [22].

### 2.7. Assay of Antifungal Activity by Agar Well Diffusion Method

All the fungal isolates were checked for purity and maintained on SDA at 4°C in the refrigerator until required for use. Antifungal activity of 1 : 1 was tested using the agar well method. Autoclaved distilled water was used for the preparation of fungal spore suspension and transferred aseptically into each SDA plate. The doses of test extracts were prepared from 5 g/100 ml stock solution from which 30 *µ*l was added to each well. All plates were incubated at 28 ± 2°C for 24–48 hr, and after incubation, the diameter of zone of inhibition was measured [23].

### 2.8. DPPH Radical Scavenging Assay

DPPH radical scavenging by *A. otallensis* plant extracts was estimated according to a previously reported method [24]. 2 ml of DPPH solution in methanol (0.004%, 0.102 mM) was mixed with 2 ml of extracts with different concentrations (200–800 mg/l). For blank solution, the extracts were substituted by methanol and used for the correction of the baseline at 515 nm. The tubes were allowed to stand at 20–25°C for 20 min. The antiradical activity was based on the measurement of the reducing ability of the plant extract towards DPPH radical. Ascorbic acid was used as a standard in the range of (25–100 mg/l), and the scavenging effects of the leaf extracts were determined with a linear curve of the ascorbic acid standard.

### 2.9. Determination of Total Phenol by Folin–Ciocalteau Reagent Method

Total phenol content was determined by the Folin–Ciocalteau reagent method with some modification. From each crude extract, 1 mg was dissolved in 1 ml of methanol. A total of 10% Folin–Ciocalteau reagent was prepared by adding the Folin–Ciocalteau reagent (10 ml) in water (90 ml). Then, 5% Na_2_CO_3_ (3 g) was prepared by dissolving Na_2_CO_3_ (3 g) in water (50 ml). 200 *µ*l of each crude extract were taken in a test tube, and 1.5 ml of 10% Folin–Ciocalteau reagent was added. Then, all the test tubes were kept in a dark place for 5 min. Finally, 1.5 ml of 5% Na_2_CO_3_ was added to the solutions and mixed well by hand. Again all the test tubes were kept in the dark for 2 hr. The absorbance was measured for all solution by using a UV spectrophotometer at a constant wavelength of 750 nm. Gallic acid was used as a standard in the range of 2.5–100 mg/l, and the phenol contents of the plant extracts were determined with a linear curve of gallic acid standard [25].

### 2.10. X-Ray Fluorescence Spectroscopy (XRF) Analysis

XRF analyses were performed using 100 g of the plant sample. The XRF measuring system consisted of a multichannel analyzer ORTECR, semiconductor detector Si/Li (thickness of beryllium window = 0.25 mm, the diameter of beryllium window = 5 mm), and radionuclide source of radiation 238 Pu (*A* = 370 MBq, *E* = 12–22 keV, *T* = 86.4 years) made by Amersham in the form of the planar disk source. All the measurements were performed in the noncoaxial geometrical arrangement of source, sample, and detector, and the acquisition time was 2000 seconds [26].

### 2.11. Data Analysis

All the experiments were performed in triplicates to minimize the experimental error, while data were reported as the mean ± SD (*n* = 3). Statistical analysis of assays results was performed using one way ANOVA, and statistical analysis was performed using the post hoc Tukey test of SPSS 25 statistical software. A *p* value less than or equal to 0.05 was adopted as the statistical significance level.

## 3. Results and Discussion

### 3.1. Percentage Yield of Extracts

The solvent methanol showed a higher degree of yield in the leaf extract which is 8.45 (22.27%), while the distilled water extract of the leaf showed less degree of yield which is 0.22 g (1.10%) as shown in Table 1. The solvent methanol showed a high degree of yield in the root extract which is 3.12 g (15.58%), while the petroleum ether extract showed less degree of yield which is 0.42 g (2.1%) as shown in Table 1. The polarity of the various compounds that are found in the plant causes variations in the extraction yield, which have been reported in the literature on medicinal plants from Vietnam [27]. To generate extracts with acceptable yields and potent antibacterial activity, solvent selection is a crucial step.

### 3.2. Phytochemical Screening

The phytochemical analysis of the *A. otallensis* gel extract demonstrates a notable presence of alkaloids, phenols, saponins, tannins, steroids, terpenoids, glycoside, anthraquinones, and phlobatannals. The methanolic extract of the root contained glycoside, diterpenes, phytosterols, and phlobatannals, while the methanolic extract of the leaf showed the presence of triterpenoids, alkaloids, tannins, and glycoside. A photochemical analysis shows that the petroleum ether extract of the root included a presence of flavonoids, saponins, glycosides, anthraquinones, diterpenes, and phytosterols, while the petroleum ether extract of the leaf contained alkaloids and anthraquinones. According to a photochemical analysis, terpenoids, anthraquinones, diterpenes, and phytosterols were remarkably found in n-hexane extract of the root, whereas alkaloids, flavonoids, saponins, terpenoids, triterpenoids, and anthraquinones were detected in the n-hexane extract of the leaf. Likewise, the photochemical analysis indicates a notable existence of saponins, glycosides, anthraquinones, and terpenes in the distilled water extract of the root, whereas the distilled water extract of the leaf contained saponins, tannins, terpenoids, and anthraquinones.  Table 2 shows the phytochemical composition of each extract.

It is known that the photochemical substances that were identified have medicinal uses. Alkaloids are one such example of a potent toxin. Numerous alkaloids that are extracted from medicinal plants exhibit biological activity such as cytotoxicity, anti-inflammatory, antimalaria, antimicrobial, and pharmacological properties [28–31]. Likewise, plant-based steroids are recognized to have cardiotonic properties in addition to their antimicrobial and insecticidal attributes. Since their biological actions are widely established, they are frequently employed in therapeutics. Research has shown that tannins possess antiviral, anticancer, and antibacterial properties [32]. Congestive heart failure and cardiac arrhythmia have been treated with additional phytochemicals known as cardiac glycosides [33]. The biological activity exhibited by *A. otallensis* and the reason behind its use in traditional medicine may be attributed to these phytochemical substances found in the gel, root, and leaf extracts.

### 3.3. Antibacterial Activity of *A. otallensis*

The result of antibacterial evaluation of the different parts of *A. otallensis* is presented in Table 3. A significance difference was observed between direct gel extract, methanol root extract, and n-hexane root extract at (*p*=0.0006) on E. *coli*. Methanol root extract showed significant relationships with direct gel extract at (*p*=0.0006), leaf methanol extract at (*p*=0.0283), leaf petroleum extract at (*p*=0.0084), and positive control (amoxicillin) at (*p*=0.0026) on E. *coli*. On the other hand, a significance difference was observed between methanol, petroleum ether, and distilled water extracts of leaf at (*p*=0.0072) on S. *aureus*. Both leaf and root extracts of methanol showed significant difference with positive control at (*p*=0.004) but no significant difference observed between direct gel extract with leaf methanol extract at (*p*=0.072) and leaf petroleum ether extract at (*p*=0.374) on S. *aureus*. Differences in antimicrobial activity of medicinal plants are obviously related to differences in their contents of active compounds [34]. Available reports tend to show that alkaloids and flavonoids are the responsible compounds for the antimicrobial activities in higher plants [35]. Moreover, it is also claimed that secondary metabolites such as tannins and other compounds of phenolic nature are classified as active antimicrobial compounds [36]. The primary bioactive components and antimicrobial agents found in plants are flavonoids, phenolic compounds, tannins, and alkaloids [37]. Some bioactive compounds inhibit microorganisms from carrying out their biological functions via altering their biochemical systems, attaching to their protein molecules, serving as chelating agents, or inducing cell inflammation [38]. According to [39], several plant extracts have been found to be ineffective against specific test organisms at lower concentrations; this may be because the extracts contain fewer antimicrobial agents. Consistent with our findings, [40] found that the *S. aureus* ATCC25923 strain is susceptible to both aqueous and ethanol extracts in their research on *Matricaria pubescens*.

### 3.4. Minimum Inhibitory Concentration (MIC) of Plant Extracts

Crude plant extract was serially diluted using 5% DMSO for the MIC test. A MIC calculation was performed for extracts that displayed a diameter of at least 6 mm within the growth inhibition zone at a concentration of 25 mg/ml. The reference bacterial strains were treated with crude extracts of direct gel extract, root, and leaf extracts with different solvents' (i.e., methanol, chloroform, n-hexane, and distilled water) results are shown in Table 4. The antibacterial activity was considered as significant when the MIC was less than 100 *μ*g/mL, moderate when the MIC was between 100 and 625 *μ*g/mL, and low when the MIC was greater than 625 *μ*g/mL [41]. The results of MIC value of direct gel extract with leaf methanol and leaf petroleum ether noted the significant difference against S. *aureus* at (*p*=0.0072) and (*p*=0.0151), respectively. The root extract of methanol and distilled water indicated positive association at (*p*=0.0465), and also, the root extract of n-hexane and leaf methanol showed positive relationship at (*p*=0.0262) against S. *aureus*. No significant difference was observed between direct gel and leaf petroleum ether extract on S. *aureus* at (*p*=0.0787). On the other hand, MIC of direct gel extract and leaf methanol extract showed a significant difference at (*p*=0.0200) and direct gel extract with leaf n-hexane extract at (*p*=0.0052) against E. *coli*, respectively. Similarly, root distilled water extract and leaf methanol extract noted a significant relationship at (*p*=0.0025) and also root distilled water extract with leaf n-hexane at (*p*=0.0005) against E. *coli*, respectively. Unfortunately, the MIC of direct gel extract with root methanol extract and leaf petroleum ether extract with root n-hexane had showed no significant difference at (*p*=0.6416) and (*p*=0.7174) on E. *coli*, respectively. The results of the MIC determination test showed that different plant parts had varying minimum concentrations of the crude extract that might inhibit the reference bacteria's growth. The plant's antibacterial activity against the test bacterium lines up with the MIC value. This finding was corresponding with the earlier report of Tadele [42], who reported 5 mg/ml against *S. aureus*. MIC that is extremely low indicates significant antibacterial activity [43]. This is consistent with our findings because [44] reported that the MIC of extracts against various strains varied depending on the strains tested and the extract in question.

### 3.5. Antifungal Activity of *A. otallensis*

Antifungal activity of the *A. otallensis* root, leaf, and gel was checked against fungi (dandruff). During antifungal activity test, a significant difference was observed between gel and root extract at the (*p*=0.0063), but there was no significant difference between leaf extract with gel and root extract at (*p*=0.17). The result as expressed in Table 5 showed that the root has no effect on fungi (dandruff). A different study reported that *Aloe* species possesses antifungal activity. Aqueous extract of *A. otallensis* can be used to treat infections from fungi (dandruff).

Our study investigated the antibacterial and antifungal activities of *Commelina diffusa Burm. F.* extracts from different plant parts using various solvents. We observed significant variations in activity against the same microorganisms, which can be attributed to differences in plant part composition and extraction solvents. Different plant parts (leaves, stems, and roots) contain varying concentrations of bioactive compounds. Extraction solvents also play a crucial role, influencing which compounds are extracted and their overall antimicrobial efficacy. For example, polar solvents like methanol extract a wider range of compounds compared to nonpolar solvents like hexane, impacting antimicrobial activity differently.

### 3.6. Antiradical Activity of Plant Extracts

**Figure 1:**
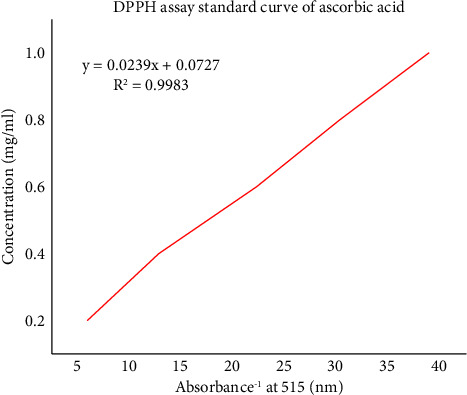
Standard curve of ascorbic acid.

The DPPH radical scavenging activities of the plant extracts were estimated by comparing the percentage scavenging activity of the DPPH with a standard, ascorbic acid Figure 1. In the present study, DPPH, a stable free radical with a characteristic absorption at 515 nm, was used to study the radical-scavenging effects. Ascorbic acid was used as a standard since ascorbic acid is considered to be a strong antiradical due to its ability to scavenge free radicals and bind transition metal ions.

**Figure 2:**
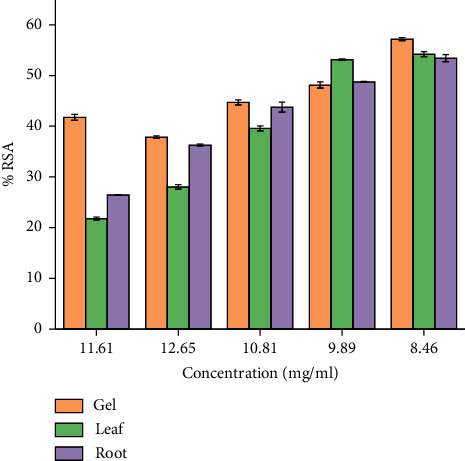
Radical scavenging activity (% RSA) of *A. otallensis* plant.

The results of DPPH free radicals scavenging are presented in Figure 2. The decrease in absorption is taken as a measure of the extent of radical scavenging. The radical-scavenging activity values were expressed as the ratio percentage of sample absorbance decrease and the absorbance of DPPH solution in the absence of extract at 515 nm. *A. otallensis* plant parts were proved to be inhibiting the DPPH free radical scavenging activity with IC_50_ value as shown in Figure 2. This means that it shows considerable antiradical activity in quenching the free radical scavenging of DPPH.

**Figure 3:**
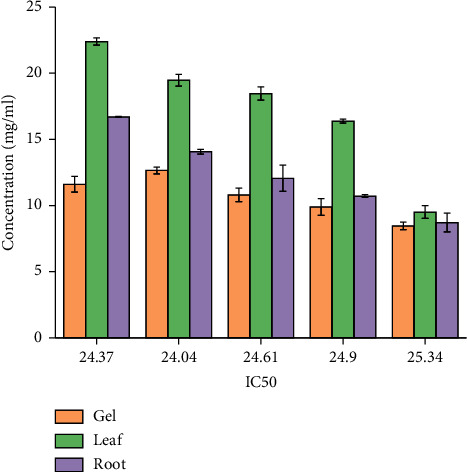
IC_50_ values of *A. otallensis* plant extracts.

In general, among all the plant extracts, the crude extracts obtained from *A. otallensis* gel extracts showed the best antiradical activity, and on the other hand, *A. otallensis* leaf and root extract shows lower antiradical activities, respectively. A correlation between the total phenolic content and antioxidant activity has been found by some authors [45]. According to [46], antioxidant substances may have potent metal chelation activities. Because of their redox characteristics, they quench singlet and triplet oxygen, donate hydrogen and function as reducing agents [47]. The extract's antioxidant content, as well as the interactions and structures of these molecules, determine its antioxidant capacity [48]. The inhibition effect of different concentrations of the plant extract and ascorbic acid in scavenging of DPPH was evaluated. The IC_50_ values of the plant extracts are presented in Figure 3.

In general, among all the plant extracts, the crude extracts obtained from *A. otallensis* gel extracts showed the highest IC_50_, and on the other hand, *A. otallensis* leaf and root crude extract shows lower IC_50_, respectively.

### 3.7. Total Phenolic Content of Plant Extracts

**Figure 4:**
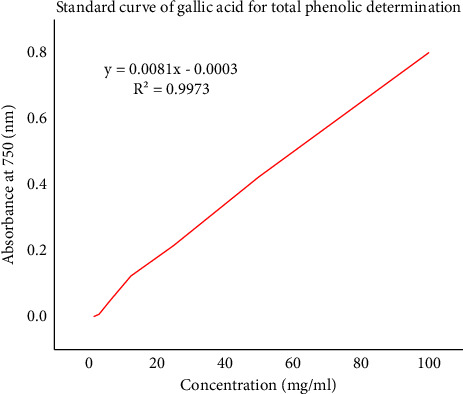
Total phenolic content for standard gallic acid, values expressed in terms of gallic acid.

The content of phenolic compounds in plant extracts of *A. otallensis* was determined from regression equation of calibration curve of gallic acid Figure 4 and expressed as milligrams, the equivalent of gallic acid per gram of dry extract (mg GAE/g) in Table 6.

The total phenolic content calculated in this study is presented in Table 6. The highest value of phenolic content was obtained in *A. otallensis* gel methanol extract followed by leaf extract, while *A. otallensis* root methanol extract shows lower phenolic content as shown in Table 6.

Strong chain-breaking antioxidants such chemical compounds called phenol are known to have direct effects on antioxidant activity [49, 50]. These phenolic compounds contribute to antioxidant activity due to the arrangement of functional groups (hydroxyl) in their nuclear structure for hydrogen donation to stabilize radical molecules [51].

### 3.8. XRF Analysis of *A. otallensis* Plant Parts

In this work, XRF spectroscopy was used to determine the percentage and concentration of different elements in gel, leaf, and root *A. otallensis* plant. In the gel extract of A. *otallensis*, the concentrations of various elements were determined as follows: potassium (K) was found to be 3482.64 ± 114.02 ppm, calcium (Ca) measured 886.71 ± 45.67 ppm, iron (Fe) was detected at 128.29 ± 16.5 ppm, titanium (Ti) at 49.08 ± 23.52 ppm, zirconium (Zr) at 9.81 ± 1.30 ppm, molybdenum (Mo) at 9.54 ± 1.41 ppm, zinc (Zn) at 8.87 ± 3.48 ppm, and strontium (Sr) at 5.35 ± 0.89 ppm, while uranium (U), chromium (Cr), vanadium (V), and scandium (Sc) were not detected (0 ppm each) as shown in Table 7.

In the leaf extract of *A. otallensis*, the concentrations of various elements were also determined as follows: Potassium (K) was found to be 3136.27 ± 105.71 ppm, Calcium (Ca) measured 2125.39 ± 61.63 ppm, Iron (Fe) was detected at 101.36 ± 26.69 ppm, Chromium (Cr) at 44.32 ± 7.85 ppm, Strontium (Sr) at 33.87 ± 1.64 ppm, Scandium (Sc) at 22.35 ± 7.36 ppm, Vanadium (V) at 15.53 ± 8.15 ppm, Zirconium (Zr) at 10.92 ± 1.63 ppm, Molybdenum (Mo) at 9.55 ± 1.67 ppm, Zinc (Zn) at 7.23 ± 4.23 ppm, Uranium (U) at 4.16 ± 2.54 ppm, and Titanium (Ti) was not detected (0 ppm) as shown in Table 7.

The root extract of *A. otallensis* was analyzed for its elemental composition, revealing significant concentrations of various elements. Calcium (Ca) was found to be the most abundant element, measuring 29657.04 ± 245.17 ppm, followed by Potassium (K) at 8667.62 ± 193.07 ppm and Iron (Fe) at 632.61 ± 33.22 ppm. Strontium (Sr) was detected at 110.17 ± 2.87 ppm, Scandium (Sc) at 105.06 ± 27.27 ppm, Titanium (Ti) at 91.61 ± 18.84 ppm, Chromium (Cr) at 61.22 ± 5.56 ppm, Zinc (Zn) at 37.02 ± 5.75 ppm, Vanadium (V) at 17.91 ± 5.97 ppm, Molybdenum (Mo) at 8.8 ± 1.82 ppm, Zirconium (Zr) at 8.32 ± 1.96 ppm, and Uranium (U) at 6.36 ± 3.03 ppm as shown in Table 7.

These findings provide insights into the elemental profile of *A. otallensis* root extract, highlighting its potential nutritional and medicinal properties based on the presence of these elements. The mineral composition of the *A. otallensis* plant revealed the presence of all the mineral elements, and the interaction of trace minerals composition present in the medicinal plants has great importance to understand their functions in the human body.

## 4. Conclusions

The choice of the extraction method and solvent must be carefully considered in order to maximize extract yield and bioactivity. The percentage yield of *A. otallensis* plant extract with various solvents has been compared, and among those solvents, methanol yields higher than other solvents of *A. otallensis* plant parts. Due to its high content of secondary metabolites, *A. otallensis* is used in traditional medicine to treat and prevent infections. The antibacterial assay employed in this work revealed that *E. coli* was more susceptible to the plant extracts than *S. aureus.* The minimum inhibitory concentration of gel showed list than root and leaf plant extract of *A. otallensis* against S. *aureus* and E. *coli*. Compared to other extracts, gel extracts exhibited superior MIC activity and strong antibacterial activity with antibiotics. The study's findings showed that the *A. otallensis* gel plant part's methanol extracts made using maceration techniques had a high antiradical and phenolic content and included a variety of three macro, eight micro, and one heavy metal components.

## Figures and Tables

**Table 1:** Percentage yield of *A. otallensis* leaf and root extracts.

Plant name	Part	Solvent	Weight of extract obtained (g)	Yield percentage (%)
*A. otallensis*	Leaf	Methanol	8.45	22.27
		Petroleum ether	3.02	15.09
		*n*-hexane	0.59	2.96
		Distilled water	0.22	1.10

*A. otallensis*	Root	Methanol	3.85	14.56
		Petroleum ether	0.45	2.25
		*n*-hexane	3.65	13.96
		Distilled water	0.42	2.10

**Table 2:** Preliminary phytochemical compounds in *A. otallensis* plant from different extracts.

No.	Photochemical analysis	Gel	Methanol		Petroleum ether		*n*-hexane		Distilled water	
			Root	Leaf	Root	Leaf	Root	Leaf	Root	Leaf
1	Alkaloid	+	−	+	−	+	−	+	−	−
2	Flavonoid	−	−	−	+	−	−	+	−	−
3	Phenols	++	−	−	−	−	−	−	−	−
4	Saponins	+	−	−	+	−	−	+	+	++
5	Tannins	++	−	+	−	−	−	−	−	+
6	Steroids	+	−	−	−	−	−	−	−	−
7	Terpenoids	+	−	−	−	−	+	+	−	+
8	Triterpenoids	−	−	+	−	−	−	+	−	−
9	Glycoside	++	+	+	+	−	−	−	+	−
10	Anthraquinones	++	−	−	+	+	+	+	+	+
11	Diterpenes	−	+	−	+	−	+	−	+	−
12	Phytosterols	−	+	−	+	−	+	−	−	−
13	Phlobatannals	+	+	−	−	−	−	−	−	−

Key: ++ = highly present, + = present, − = absent.

**Table 3:** Antibacterial activity of *A. otallensis* plant extracts.

Organism	Plant part	Solvent	Zone of inhibition (mm)
*Escherichia coli*	Gel	Direct extract	28 ± 4.51
	Leaf	Methanol	18 ± 5.00
		Petroleum ether	20 ± 2.00
		*n*–hexane	13 ± 6.00
		Distilled water	15 ± 2.00
	Root	Methanol	−
		Petroleum ether	15 ± 2.00
		*n*–hexane	−
		Distilled water	11 ± 3.00
		Ampicillin	23 ± 5.51

*Staphylococcus aureus*	Gel	Direct extract	14 ± 2.00
	Leaf	Methanol	−
		Petroleum ether	18 ± 3.00
		*n*-hexane	10 ± 3.00
		Distilled water	−
	Root	Methanol	15 ± 2.00
		Petroleum ether	−
		*n*-hexane	12 ± 4.00
		Distilled water	−
		Ampicillin	19 ± 2.52

Key: − = absent.

**Table 4:** Minimum inhibitory concentrations of crude extracts of *A. otallensis* (mg/ml).

Plant part	Solvents	MIC of crude extracts (mg/ml)^a^	
		*S. auras*	*E. coli*
Gel	Direct extract	1.23 ± 0.40	1.34 ± 0.30

Root	Methanol	3.11 ± 0.40	2.25 ± 0.30
	Petroleum ether	3.08 ± 0.40	4.26 ± 0.20
	*n*-hexane	2.10 ± 0.20	3.60 ± 0.30
	Distill water	_b	_b

Leaf	Methanol	5.56 ± 0.30	4.85 ± 0.25
	Chloroform	3.87 ± 0.40	3.20 ± 0.20
	*n*-hexane	5.30 ± 0.20	5.58 ± 0.30
	Distill water	_b	_b

Key: a = mean values from triplicate, _b = No MIC.

**Table 5:** Antifungal activities of *A. otallensis*.

Test organism	Diameter of zone of inhibiting (mm) of *A. otallensis*		
	Leaf (mm)	Gel (mm)	Root
Dandruff	9 ± 3.0	17 ± 2.0	—

**Table 6:** Total phenolic content (mg GAE/g) of methanol extract of *A. otallensis*.

Solvent	Parts used	Total phenolic content (mg GAE/g)
Methanol	Gel	61.9 ± 0.5
Methanol	Leaf	53.6 ± 0.3
Methanol	Root	51.6 ± 0.6

**Table 7:** Evaluation of elemental content of *A. otallensis* plant by X-ray fluorescence spectroscopy.

S. No-	Gel		Leaf		Root	
	Element	Concentration (ppm)	Element	Concentration (ppm)	Element	Concentration (ppm)
1	Ca	886.71 ± 45.67	Ca	2125.39 ± 61.63	Ca	29657.04 ± 245.17
2	Cr	0	Cr	44.32 ± 7.85	Cr	61.22 ± 5.56
3	Fe	128.29 ± 16.5	Fe	101.36 ± 26.69	Fe	632.61 ± 33.22
4	K	3482.64 ± 114.02	K	3136.27 ± 105.71	K	8667.62 ± 193.07
5	Mo	9.54 ± 1.41	Mo	9.55 ± 1.67	Mo	8.8 ± 1.82
6	Sc	0	Sc	22.35 ± 7.36	Sc	105.06 ± 27.27
7	Sr	5.35 ± 0.89	Sr	33.87 ± 1.64	Sr	110.17 ± 2.87
8	Ti	49.08 ± 23.52	Ti	0	Ti	91.61 ± 18.84
9	U	0	U	4.16 ± 2.54	U	6.36 ± 3.03
10	V	0	V	15.53 ± 8.15	V	17.91 ± 5.97
11	Zn	8.87 ± 3.48	Zn	7.23 ± 4.23	Zn	37.02 ± 5.75
12	Zr	9.81 ± 1.30	Zr	10.92 ± 1.63	Zr	8.32 ± 1.96

## Data Availability

All data and materials are mentioned in the paper.
